# Lactate from astrocytes fuels learning-induced mRNA translation in excitatory and inhibitory neurons

**DOI:** 10.1038/s42003-019-0495-2

**Published:** 2019-07-02

**Authors:** Giannina Descalzi, Virginia Gao, Michael Q. Steinman, Akinobu Suzuki, Cristina M. Alberini

**Affiliations:** 1 Center for Neural Science, New York University, New York, NY 10003 USA; 2 Present Address: Department of Biochemistry, Faculty of Medicine, Graduate School of Medicine & Pharmaceutical Sciences, University of Toyama, 2630 Sugitani, Toyama, 930-0194 Japan

**Keywords:** Learning and memory, Consolidation, Neuroscience, Astrocyte

## Abstract

Glycogenolysis and lactate transport from astrocytes to neurons is required for long-term memory formation, but the role of this lactate is poorly understood. Here we show that the Krebs cycle substrates pyruvate and ketone body B3HB can functionally replace lactate in rescuing memory impairment caused by inhibition of glycogenolysis or expression knockdown of glia monocarboxylate transporters (MCTs) 1 and 4 in the dorsal hippocampus of rats. In contrast, either metabolite is unable to rescue memory impairment produced by expression knockdown of MCT2, which is selectively expressed by neurons, indicating that a critical role of astrocytic lactate is to provide energy for neuronal responses required for long-term memory. These responses include learning-induced mRNA translation in both excitatory and inhibitory neurons, as well as expression of Arc/Arg3.1. Thus, astrocytic lactate acts as an energy substrate to fuel learning-induced de novo neuronal translation critical for long-term memory.

## Abstract (web summary)

Descalzi et al. show that pyruvate and ketone body B3HB rescue the memory deficits caused by the glia-specific knockdown of monocarboxylate transporters in the rat hippocampus. Astrocytic lactate is also required for learning-induced de novo protein synthesis in excitatory and inhibitory neurons, highlighting its importance in memory consolidation.

## Introduction

Glucose metabolism regulation is critical for most fundamental cellular processes and is tailored according to the demands of specific cell types. In the brain, glucose provides the major source of energy via its direct glycolysis or can be stored as glycogen. Glycogen can be rapidly catabolized through glycogenolysis and anaerobic glycolysis into pyruvate and lactate in response to increases in energy demands^1^. Among the cells in the brain, neurons have the highest energy needs, and astrocytes, which are the predominant storage sites of glycogen^2–4^, can supply lactate to neurons in an activity-dependent manner^5,6^. Memory formation, as well as long-term synaptic potentiation (LTP), requires glycogenolysis and astrocytic–neuronal lactate transport^7–13^. More specifically, in rodents, learning is accompanied by a glycogenolysis-dependent increase of lactate in the extracellular space of the hippocampus. Inhibition of this process prevents memory formation, synaptic plasticity, and underlying molecular changes^7,10,12^. Moreover, studies employing functional inhibition or knockdown of astrocytic or neuronal monocarboxylate transporters (MCTs), through which lactate can bidirectionally travel, provided evidence that lactate transport from astrocytes to neurons is required for memory formation and drug-induced conditioned responses^7,10,12^. Nevertheless, the target mechanisms within neurons that demand learning-regulated lactate supply remain unknown. In neurons, lactate may exert multiple, non-mutually exclusive critical roles important for memory formation, including providing energy via the Krebs cycle, contributing to redox regulation, or activate cell signaling through the receptor, GPR81/HCAR1^6,14–19^.

Furthermore, although experimental support exists for the transfer of lactate from astrocytes to neurons, the role of lactate versus glucose as neuronal energy substrates is debated^5,20–28^. As there is evidence that in the retina a neuron to glia lactate shuttling exists, the functional significance and direction of lactate shuttling in vivo is also debated^5,22–28^. In this study, we investigated whether lactate generated from glycogenolysis, therefore astrocytes, and shuttled into neurons via MCT2, acts as an energy substrate to fuel the high energy demands of neuronal processes underlying long-term memory formation. Specifically, we tested whether lactate fuels energy required for learning-induced de novo protein synthesis, a hallmark mechanism required for long-term memory formation, and one of the most energy-intensive cellular processes^29–32^, whose rate strongly correlates with cellular metabolic activity^33,34^. To test this hypothesis, we employed the contextual fear-based inhibitory avoidance (IA) task in adult rats and focused on the dorsal hippocampus (dHP), a brain region critical for long-term episodic memory formation.

## Results

### Pyruvate or B3HB can replace glycogenolysis and lactate in memory formation

**Fig. 1:**
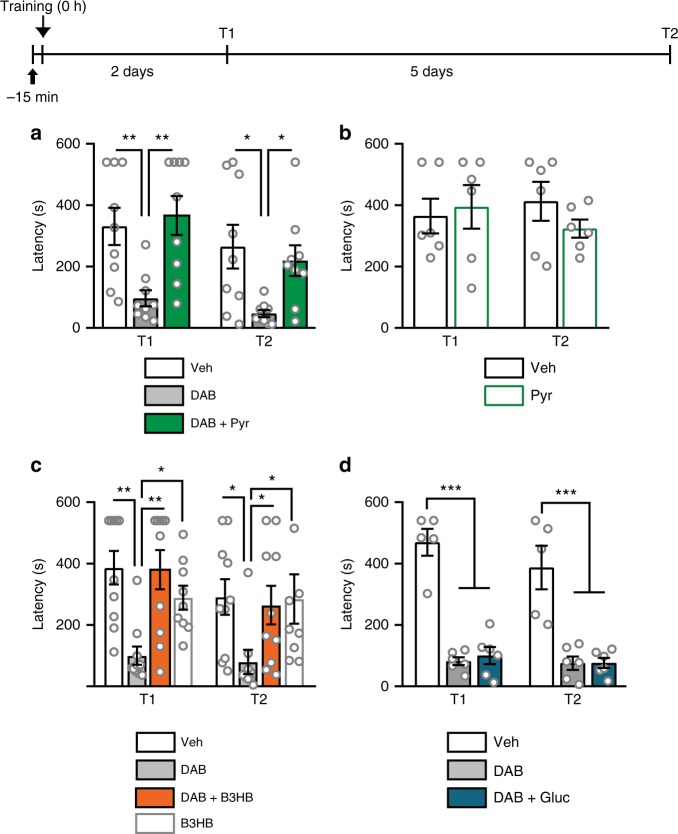
Memory impairments caused by blockade of glycogenolysis are rescued by pyruvate or B3HB. Memory retention expressed as mean latency ± SEM (in seconds, s). Timeline is presented above graphs. **a** A bilateral intra-dHP injection of vehicle (Veh), DAB (300 pmol), or DAB + pyruvate (Pyr) (100 nmol), was given 15 min before IA training (black arrow), and IA memory retention was tested 2 (T1) and 7 days (T2) after training. *n* = 9/group, two-way RM ANOVA, SNK; 4 independent experiments. **b** A bilateral intra-dHP injection of vehicle (Veh) or pyruvate (Pyr) was given 15 min before IA training (black arrow), and IA memory retention was tested 2 (T1) and 7 days (T2) after training. *n* = 6/group, two-way RM ANOVA; 3 independent experiments. **c** A bilateral intra-dHP injection of vehicle (Veh, *n* = 10), DAB (300 pmol, *n* = 10), DAB + B3HB (72 nmol, *n* = 10), or B3HB (*n* = 9) alone was given 15 min before IA training, and IA memory retention was tested 2 (T1) and 7 days (T2) after training. Two-way RM ANOVA, SNK; 6 independent experiments. **d** A bilateral intra-dHP injection of vehicle (Veh, *n* = 5), DAB (*n* = 6), or DAB + glucose (Gluc, 50 nmol, *n* = 6), was given 15 min before IA training (black arrow), and IA memory retention was tested 2 (T1) and 7 days (T2) after training. Two-way RM ANOVA, SNK. 3 independent experiments. *p < 0.05, **p < 0.01, ***p < 0.001. See Supplementary Table 1 for detailed statistical information

To determine whether lactate shuttling into neurons is an essential source of energy for neuronal processes required for long-term memory formation, we tested whether supplying glycolytic metabolites downstream of lactate, and specifically pyruvate or the ketone body β-hydroxybutyrate (B3HB), would be sufficient to rescue memory impairment caused by inhibition of glycogenolysis or astrocytic lactate transport. Both substrates are transported into neurons via MCT2, and readily enter the Krebs cycle to produce energy in the form of ATP^35,36^. We reasoned that if lactate plays a critical role as an energy substrate in long-term memory formation, then either pyruvate or B3HB would be sufficient to functionally replace it. We found that, as expected, bilateral dHP injections of 1,4-dideoxy-1,4-imino-D-arabinitol (DAB; 300 pmol), an inhibitor of glycogen phosphorylase and synthase activities that blocks learning-induced astrocytic lactate production, 15 min prior to IA training disrupted long-term memory formation in adult rats^7^. Co-injection of DAB with equicaloric concentration of pyruvate (100 nmol) or B3HB (72 nmol) fully rescued memory impairment (Fig. 1a–c; pyruvate: F_2,24 = _9.24, *p* = 0.001; B3HB: F_3,35_ = 5.52, *p* = 0.003; two-way repeated measures (RM) analysis of variance (ANOVA), followed by Student Newman-Keuls (SNK) post-hoc analyses). Injections of pyruvate or B3HB without DAB, compared to vehicle solution, did not affect memory retention (*p* = 0.584 and *p* = 0.933 Fig. 1b, c, respectively).

In contrast, 50 nmol of glucose (equicaloric concentration to lactate, pyruvate, or B3HB), which can enter astrocytes or neurons via the glucose transporters GLUT1 or GLUT3, respectively^37^, co-injected with DAB 15 min prior to training, was unable to rescue memory deficits (Fig. 1d; *p* = 0.768). These data strongly suggest that lactate derived from astrocytic glycogenolysis provides neurons with a critical energy substrate necessary for long-term memory formation.

### Pyruvate or B3HB rescues memory loss caused by MCT1/MCT4 but not MCT2 knockdown

To further confirm that glycolytic metabolites can serve as an energy source in neurons when lactate transport from astrocytes is blocked, we tested the effects of supplying pyruvate following expression knockdown of the astrocytic MCTs, MCT1 and MCT4. MCT1 is expressed in astrocytes, ependymocytes, oligodendrocytes, and endothelial cells of blood vessels, whereas MCT4 is mainly expressed by astrocytes and is enriched at synaptic sites^7,38,39^. MCTs bidirectionally shuttle monocarboxylates (lactate, pyruvate, and ketone bodies); therefore, reductions in MCT1 and MCT4 levels inhibits the transport of these molecules in and out of astrocytes, and for MCT1 also of the other types of cells mentioned. To avoid regulatory compensation, which can occur in genetic or virus-mediated knockdowns, we employed a temporally limited and anatomically restricted expression knockdown by using an antisense oligodeoxynucleotide (AS-ODN) approach, which has been employed successfully and validated in numerous studies^40^.

**Fig. 2:**
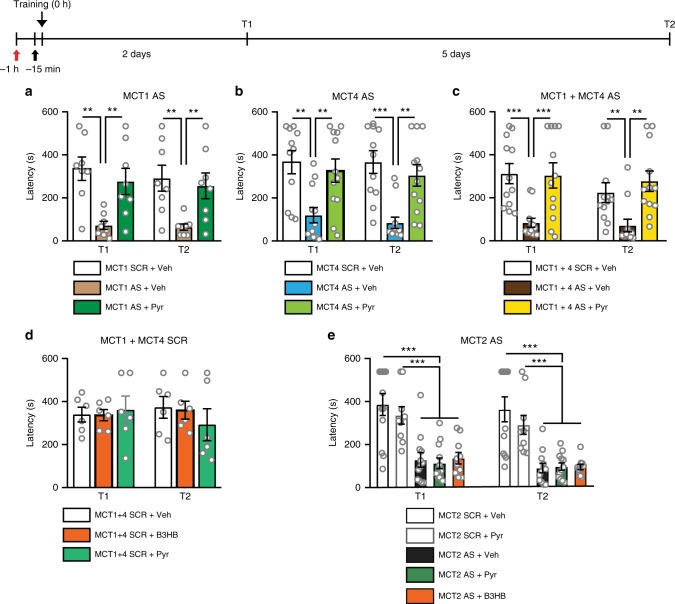
Memory impairments caused by expression knockdown of MCT1 and MCT4, but not of MCT2, are rescued by pyruvate or B3HB. Memory retention expressed as mean latency ± SEM (in seconds, s). Timeline is presented above graphs. Rats received a bilateral intra-dHP injection of related scrambled (SCR) oligodeoxynucleotide (ODNs) sequences, or antisense (AS) ODNs against MCT1 (**a**, *n* = 8), MCT4 (**b**, *n* = 11), or MCT1 + MCT4 (**c**, *n* = 12), 1 h before training (red arrow), and (**a**–**c**) vehicle (Veh, **a**: *n* = 8, **b** and **c**: *n* = 12) or pyruvate (Pyr; 100 nmol, **a**: *n* = 8, **b** and **c**: *n* = 12) 15 min before training (black arrow). Two-way RM ANOVA, SNK; 4 independent experiments. **d** Memory tested 2 days (T1) and 7 d (T2) after training in animals that received bilateral dHP injection of MCT1 + 4 SCR-ODNs (SCR) 1 h before training (red arrow), and bilateral injection of vehicle, B3HB, or pyruvate (Pyr), 15 min before training (black arrow). *n* = 6 rats/group, two-way RM ANOVA; 3 independent experiments. **e** Memory tested 2 days (T1) and 7 d (T2) after training in animals that received bilateral dHP injection of MCT2-SCR-ODNs (SCR) or MCT2-AS ODNs 1 h before training (red arrow) and bilateral injection of vehicle (Veh, *n* = 12), pyruvate (Pyr; SCR: *n* = 10, AS: *n* = 12), or B3HB (*n* = 10) 15 min before training (black arrow). Six independent experiments; Two-way RM ANOVA, SNK. **p < 0.01, ***p < 0.001. See Supplementary Table 2 for detailed statistical information

We previously showed that a bilateral dHP injection of AS-ODNs against MCT1 and/or MCT4 one hour before training down-regulates levels of the corresponding transporter relative to scrambled ODN (SCR-ODN) controls^7^. Here, using the same ODN sequences and experimental schedule, we found that a bilateral hippocampal injection of AS-ODN against either MCT1 or MCT4 persistently disrupted long-term memory, and this effect was fully reversed by bilateral dHP injection of pyruvate 15 min prior to training (Fig. 2a–c; MCT1 AS: F_2,21_ = 8.45, *p* = 0.002; MCT4 AS: F_2,32_ = 10.63, *p* < 0.001; MCT1 and 4 AS: F_2,32 _= 9.58, *p* < 0.001, two-way RM ANOVA, followed by SNK post-hoc analyses). As when injected alone, neither pyruvate nor B3HB affected memory retention when co-administered with SCR-ODN controls against MCT1 or MCT4. In fact, the pyruvate- or B3HB-injected rats displayed similar latencies as rats injected with MCT1 or MCT4 SCR-ODN and vehicle solution (*p* = 0.860; Fig. 2d). These results further supported the conclusion that the concentrations of pyruvate and B3H3 used do not affect memory retention.

Collectively, these data indicated that, when astrocytic transport of monocarboxylates is blocked, administration of the Krebs cycle substrate pyruvate is sufficient to support long-term memory formation, suggesting the hypothesis that pyruvate, which can enter neurons via MCT2, provides neurons with the necessary energy to form long-term memory. MCT2 is selectively expressed by neurons, but not glia, and transports the monocarboxylates lactate, pyruvate, and ketone bodies into and out of neurons^38,39^.

If this hypothesis is correct, disrupting the expression of MCT2 should impede long-term memory rescue by pyruvate or B3HB. Previously, we showed that a bilateral injection of MCT2-AS-ODN into the dHP one hour before training, compared to SCR-ODN, downregulates MCT2 protein levels^7^. Here we used the same ODNs and approach and found that knockdown of MCT2 with AS-ODN persistently disrupted long-term IA memory at 2 and 7 days after training, compared to SCR-ODN (Fig. 2e; F_4,51_ = 15.39, *p* < 0.001, two-way RM ANOVA, followed by SNK post-hoc analysis). In contrast to what we found with the knockdown of MCT1 and MCT4, neither pyruvate nor B3HB could rescue memory impairment induced by MCT2-AS-ODN (pyruvate, *p* = 0.916; B3HB, *p* = 0.898). Furthermore, as with MCT1 and MCT4 SCR-ODNs, co-injection of pyruvate with MCT2 SCR-ODN did not change memory retention compared to injection of MCT2 SCR-ODN in vehicle solution (*p* = 0.2), indicating that the concentration of pyruvate used does not alter memory retention. We therefore conclude that lactate produced by astrocytes plays a role in long-term memory formation by supplying neurons with energy.

### Learning-dependent translation in excitatory and inhibitory neurons requires lactate

We next investigated whether one of the highest energy-consuming cellular processes, required for long-term memory formation, the learning-induced de novo protein synthesis- is supported by lactate. In mammalian cells, mRNA translation alone consumes ∼20% of cellular ATP, not considering the energy required for biosynthesis of the translational machinery (e.g., ribosome biogenesis^33,34^). Translation rates increase in response to learning and stimuli that induce long-term plasticity^29–32^. Hence, we investigated whether this translation uses energy provided by astrocytic glycogenolysis and lactate transport into neurons.

To this end, we assessed the relative levels of active translation in the dHP of rats by performing in vivo SUrface SEnsing of Translation (SUnSET), a technique that measures active protein synthesis. This technique assesses, via western blot or immunohistochemistry, the levels of puromycin incorporation into elongating peptide chains^41^, and has been widely used to measure active in vivo translation^42–46^.

**Fig. 3:**
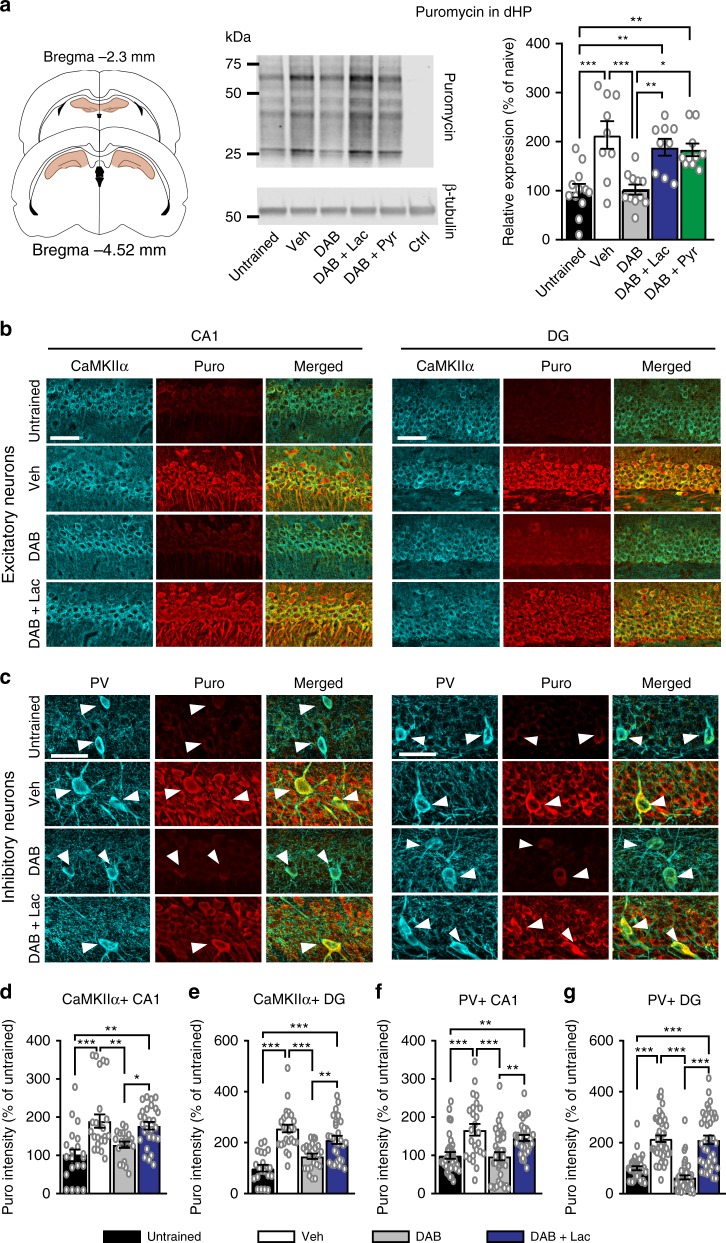
Lactate or pyruvate support learning-induced mRNA translation in both inhibitory and excitatory neurons. Data are expressed as mean % ± SEM of untrained controls (100%). SUnSET in the dHP of untrained and trained rats and its dependence on glycogenolysis and lactate. **a** Puromycin was co-injected with vehicle (Veh, naive, *n* = 12, trained *n* = 9), DAB (300pmol, *n* = 11), DAB + L-lactate (at 100 nmol, *n* = 9), or pyruvate (Pyr at 100 nmol, *n* = 10) 15 min before training. Two hours after training, dHP protein extracts were assessed for puromycin labeling via western blotting; β-tubulin was used as loading control. A control sample not injected with Puro (Ctrl) was included. Four independent experiments. See Supplementary Figure 1 for a representative SUnSET full western blot. One-way ANOVA, Tukey. **b**–**g** A dHP bilateral injection of puromycin with vehicle (Veh), DAB, DAB + L-lactate (DAB + Lac), or DAB + pyruvate (DAB + Pyr) 15 min before training. Rats were perfused 2 h after training. Brain slices were co-immunostained for puromycin (Puro) and **b** CaMKIIα or **c** parvalbumin (PV); Three independent experiments. **b**, **c** representative immunostaining examples and **d**, **e** relative quantifications expressed in mean % ± SEM of untrained controls (100%) of CaMKIIα in **d** the dHP CA1 (Untrained: *n* = 21, Veh: *n* = 25, DAB: *n* = 20, DAB + Lac: *n* = 27) and **e** dHP DG (Untrained: *n* = 21, Veh: 27, DAB: 23, DAB + Lac: *n* = 27); Three independent experiments. **f**, **g** Relative quantifications expressed in mean % ± SEM of untrained controls (100%) of PV + cells in **f** dHP CA1 (Untrained: *n* = 21, Veh: *n* = 25, DAB: *n* = 20, DAB + Lac: *n* = 27) and **g** dHP DG (Untrained: *n* = 33, Veh: *n* = 45, DAB: *n* = 45, DAB + Lac: *n* = 38); Three independent experiments. One-way ANOVA, Tukey. *p < 0.05, **p < 0.01, ***p < 0.001. Scale bars = 50 µM; see Supplementary Table 3 for detailed statistical information

Rats were subjected to bilateral dHP co-injections of puromycin (50 µg) with either vehicle, DAB (300pmol), DAB + L-lactate (100 nmol), or DAB + pyruvate (100 nmol), and dHP protein extracts were obtained 2 h after IA training, followed by measures of puromycin incorporation through western blotting. IA training resulted in increase in puromycin incorporation, hence indicating an increase in active mRNA translation (Fig. 3a). This increase was completely blocked when glycogenolysis was inhibited with DAB, but preserved if either L-lactate or pyruvate were co-administered with DAB prior to training (F_4,45_ = 10.06, *p* < 0.001; one-way ANOVA, followed by Tukey post-hoc analysis).

Studies thus far have shown that learning leads to an increase in the translation of mRNAs expressed in excitatory neurons; however, it remains to be determined whether learning-induced de novo translation also occurs in inhibitory neurons. Hence, we next quantified learning-induced de novo mRNA translation in excitatory and inhibitory neurons and asked whether lactate fuels the energy requirement for de novo protein synthesis in both neuronal types. To this end, we used double staining in immunohistochemistry (IHC) against puromycin in SUnSET and either Ca^++^/calmodulin-dependent protein kinase II alpha (CaMKIIα), a marker of excitatory neurons^47^, or parvalbumin (PV), a marker of a major subpopulation of inhibitory neurons^48^. Relative quantifications were done 2 h after training, and, in parallel, in untrained control rats. As shown in Fig. 3, IA training robustly enhanced mRNA translation in both excitatory (CaMKIIα-positive) and inhibitory (PV-positive) neurons in the CA1 and dentate gyrus (DG) subregions (Fig. 3b–g); CamKIIα: CA1 (F_3,94_ = 9.79, *p* < 0.001; one-way ANOVA, followed by Tukey post-hoc analysis), and DG (F_3, 102_ = 23.93, *p* < 0.001; one-way ANOVA, followed by Tukey post-hoc analysis); PV: CA1 (F_3,193_ = 15.89, *p* < 0.001; one-way ANOVA, followed by Tukey post-hoc analysis) and DG (F_3,157 = _42.57, *p* < 0.001; one-way ANOVA, followed by Tukey post-hoc analysis).

Moreover, inhibiting glycogenolysis with DAB 15 min prior to IA training blocked learning-induced increases in the SUnSET signal in both excitatory and inhibitory neurons. Co-injection of L-lactate with DAB completely fully rescued the levels of puromycin incorporation in both cell types, restoring their levels to those of vehicle-injected trained rats (Fig. 3d–g). Therefore, we conclude that lactate is required for the learning-induced de novo protein synthesis that takes place in both excitatory and inhibitory neurons.

Long-term memory formation requires temporally regulated translation of specific mRNAs^29–32^. To establish a link between the learning-induced mRNA translation in neurons, for which glycogenolysis and astrocytic lactate are necessary, and a target mRNA that is rapidly translated in excitatory neurons following learning, we analyzed the expression of activity-regulated cytoskeleton-associated protein (Arc/Arg3.1), an immediate early gene that plays a critical role in memory formation^49^. Arc/Arg3.1 mRNA is rapidly translated in the soma and dendrites of activated neurons; Arc/Arg3.1 transcription, mRNA transport to synapses and translation in brain regions including the hippocampus are necessary for memory formation^49^. Previously, using western blot analyses, we showed that Arc/Arg3.1 induction requires glycogenolysis and lactate^7^. Here, we sought to increase our understanding of the subregional localization of learning-induced hippocampal expression of Arc/Arg3.1, as well as tested its dependence on glycogenolysis and lactate as an energy substrate. To this end, we employed immunohistochemistry to measure the levels of Arc/Arg3.1 in the dHP 1 hour after IA training in rats treated with DAB in the presence or absence of lactate, or pyruvate, and compared them to controls trained or untrained injected with vehicle solution.

**Fig. 4:**
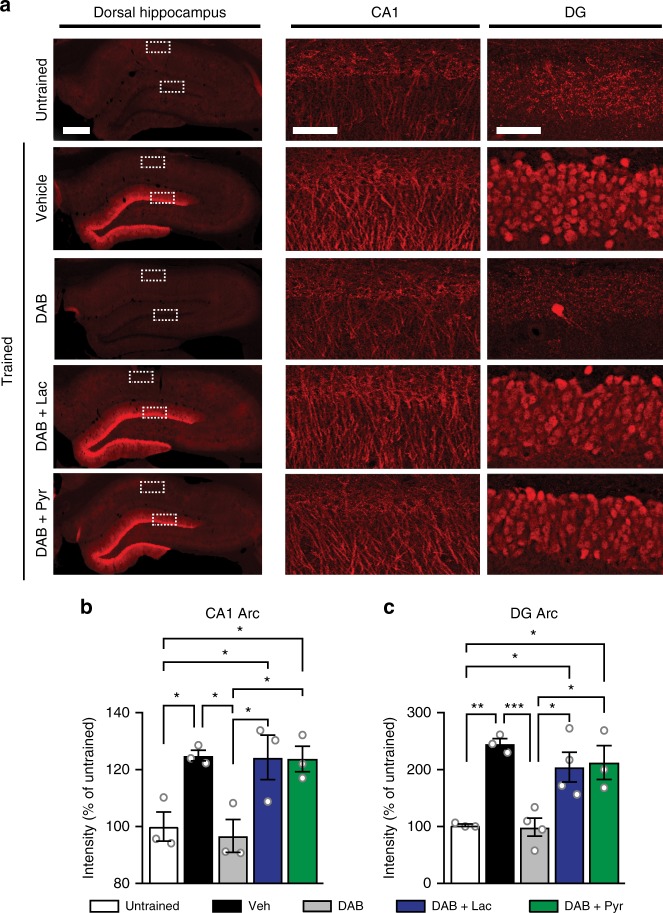
Lactate or pyruvate rescues the disruption of Arc/Arg3.1 expression caused by DAB. Representative examples **a** and relative quantifications **b** of Arc/Arg3.1 immunostaining in the dHC of untrained and trained rats injected as described. Data are expressed as mean % ± SEM of untrained controls (100%). A bilateral dHP injection of vehicle (Veh), DAB (300 pmol), DAB + L-lactate (Lac, 100 nmol), or DAB + pyruvate (Pyr, 100 nmol) was given 15 min before training, and the rats were perfused 1 h after training and compared to untrained rats injected with Veh. **a** Fluorescent images of Arc/Arg3.1 immunostaining in the dorsal hippocampus (left, scale bar = 500 µM), and higher magnifications (confocal images) of CA1 (middle, scale bar = 50 µM), and DG (right, scale bar = 50 µM). **b** Relative quantification of Arc/Arg3.1 immunohistochemical staining in CA1 (*n* = 3 rats /group; 3 brain slices per rat); 3 independent experiments. **c** Relative quantification of Arc/Arg3.1 immunohistochemical staining in DG (Untrained: *n* = 3, Veh: *n* = 3, DAB: *n* = 4, DAB + Lac: *n* = 4, DAB + Pyr: *n* = 3; 3 brain slices per rat); 3 independent experiments. One-way ANOVA, Tukey. *p < 0.05, **p < 0.01, ***p < 0.001. See Supplementary Table 4 for detailed statistical information

In vehicle-injected rats, training led to a significant and widespread increase in Arc/Arg3.1 protein expression in both the CA1(Fig. 4a, b; F_4,10_ = 8.6; *p* = 0.003, one-way ANOVA, followed by Tukey post-hoc analysis) and DG (Fig. 4a, c; F_4,13_ = 12.05; *p* < 0.001, one-way ANOVA, followed by Tukey post-hoc analysis), with a noticeably higher level of induction in the DG. Bilateral hippocampal injections of DAB completely blocked the induction of Arc/Arg3.1 expression, and returned the protein levels to those in untrained, vehicle-injected control rats (CA1: *p* = 0.984; DG: *p* = 1.0). In contrast, when DAB was co-injected with either L-lactate or pyruvate, the Arc/Arg3.1 levels were the same as those in trained, vehicle-injected rats. Pyruvate was as effective as L-lactate at restoring Arc/Arg3.1 levels following inhibition of glycogenolysis (CA1: *p* = 0.107; DG: *p* = 0.999). These results indicate that lactate acts as an energy substrate for Arc/Arg 3.1 induction evoked by learning throughout the various hippocampal excitatory neuronal populations.

## Discussion

Collectively, our data showing that pyruvate or B3HB are sufficient in replacing the critical role of glycogenolysis and lactate in long-term memory formation, suggest that one critical role of lactate required for long-term memory is to supply neurons with an essential energy substrate to fuel their high energy demands triggered by learning.

We showed that one of the neuronal high energy consuming processes that requires lactate as a source of energy is the learning-induced de novo mRNA translation, which occurs in both excitatory neurons and PV+ inhibitory neurons. Whereas activity-dependent de novo translation had been largely investigated in excitatory neurons, learning-induced de novo translation in inhibitory neurons has yet to be studied. While we found that Arc/Arg3.1 is one of the proteins rapidly synthesized in excitatory neurons that depend on energy substrates, proteins upregulated in inhibitory neurons remain to be determined. Future work is needed to gain comprehensive profiles of the translated proteins in inhibitory and excitatory neuronal populations in response to learning.

Translation is one of the highest energy consuming cellular processes, and it is a key biochemical hallmark of long-term memory^50^. Although we showed that learning-dependent translation requires lactate, we do not exclude that other processes that require fast availability of energy also depend on lactate. Nevertheless, our data support the idea that the translational control underlying synaptic plasticity and cognitive functions can be thought as a coupled astrocyte-neuronal mechanism. Furthermore, as several human cognitive disorders, including autism spectrum disorder and neurodegenerative diseases, are associated with impaired mRNA translation^51,52^, we suggest that some of these diseases may be due to alterations of astrocytic-neuronal metabolic coupling. This hypothesis has important implications for the therapeutic potential of targeting dysregulated translational control to treat cognitive disorders.

We also observed that neither pyruvate nor B3HB, when administered in control conditions, at equicaloric concentrations to those of lactate, affects memory retention. These results are consistent with those of Krebs and Parent^53^ showing that a similar concentration of pyruvate injected into the hippocampus in adult rats had no effect on spontaneous alternation, although it reversed alternation deficits produced by septal muscimol infusions. Our data, however, do not exclude the possibility that higher concentrations, or chronic treatments of pyruvate or B3HB, may produce memory-enhancing effects. Evidence in fact shows that chronic treatment with the ketone body 3-HB derivative, 3-hydroxybutyrate methyl ester, enhances memory retention in a water maze test^54^. In addition, chronic increase of ketone bodies through ketogenic diets (diets consisting of little to no carbohydrates and stimulate endogenous ketogenesis), appears to have beneficial effects on memory impairments, such as those found in Alzheimer’s disease^55,56^.

In addition, the lack of pyruvate or B3HB effects on memory retention when administered alone is similar to what we previously found with equicaloric concentrations of lactate^7,57^. In contrast, Newman et al.^10^ showed that a bilateral lactate injection at a similar concentration into the ventral hippocampus enhances spontaneous alternation. This is a spatial exploration task, which assesses working memory–a form of short-term memory. The differential effect of lactate on spontaneous alternation *vs*. long-term episodic memory tested in our experiments may be due to various reasons, including distinct brain region targeted, functional kinetics, and/or energy demands. While we targeted the dorsal hippocampus, Newman et al.^10^ injected lactate into the ventral hippocampus. Moreover, the effects of lactate on spontaneous alternation were tested right after the injection, when lactate was likely still on board when retention was assessed, whereas our IA long-term memory was tested 24 h and 7 days after training, hence in the absence of the increased lactate^7,57^. Finally, long-term and short-term memories are different memory processes, with the highly energy consuming process of mRNA translation being a distinctive mechanism required only for long-term memory.

The role of aerobic glycolysis in providing the energy necessary for learning-induced mRNA translation in neurons is intriguing and raises the general question of why aerobic glycolysis is engaged in activity-dependent brain functions. We speculate that glycogenolysis and aerobic glycolysis are engaged in long-term memory formation as well as other brain functions associated with rapid neural activation in physiological conditions because they maintain homeostasis of glycolytic intermediates and ATP during large shifts in glucose supply or demand^58^. In this regard, it is also interesting to note that glucose injected at equicaloric concentration to lactate was unable to reverse memory deficits evoked by DAB, which is in agreement with previous results showing that while lactate reversed memory deficits imposed by knockdown of MCT1 or MCT4, equicaloric concentration of glucose failed to do so^7^.

Why is glucose unable to replace lactate? If glycolysis is the critical main mechanism involved, glucose should have effects similar to lactate. One explanation may reside in the timescale of the rapid energy demands imposed by learning, which would be in agreement with the fact that glycogen, rather than exogenous glucose entering the system, is utilized. The temporal comparison of the effect of blocking glycogenolysis via DAB injected before *vs*. immediately after learning suggests that astrocytic glycogenolysis is very rapidly recruited and preferentially used over glucose import. In fact, while 300 nmol of DAB injected before training disrupts long-term memory, the same DAB concentration injected immediately after training has no effect at 24 h and much less effect at later retention times^7^. Glycogen, present in subcellular compartments close to synapses can be rapidly mobilized, hence more readily available and at less costs than glucose delivered via the blood stream^4,59^. Thus, it appears that astrocytic glycogenolysis allows for fast and localized lactate production, which is promptly transported to activated neurons to readily support the high energy demands needed for changes necessary for memory consolidation. Notably, in agreement with our data showing the necessary role of glycogenolysis and lactate transport into neurons to fuel neuronal de novo translation evoked by learning, the rapid kinetic requirement for de novo translation in memory consolidation mirrors that of glycogenolysis^60^.

We also found that pyruvate and B3HB, like lactate, are sufficient to reverse the memory deficits produced by blocking glycogenolysis only when their entry into neurons is possible. This result is in line with the conclusion that the directionality of lactate transport goes from astrocytes to neurons.

Given the fact that lactate can also shuttle out of neurons, as MCTs bi-directionally transport monocarboxylates, we have considered whether our data could be explained by learning-induced neuronal-astrocytic shuttling. However, this explanation was rejected because: (1) Antisense-mediated knockdown of MCT1 or MCT4 is sufficient to disrupt memory formation indicating that extracellular lactate originating from neurons is not sufficient to support neuronal processes involved in memory formation, and (2) Expression knockdown of MCT2 impairs memory, and, in this case, pyruvate and B3HB, like lactate^7^, are unable to rescue memory, whereas memory impairments caused by knockdown of MCT1 and/or MCT4 are rescued by pyruvate, just like with lactate^7^. Collectively, these results, together with the evidence that lactate, pyruvate, and B3HB also rescue memory impairments caused by glycogenolysis disruption lead us to conclude that lactate must exit astrocytes and enter neurons where it provides a critical role for memory formation. This conclusion is also in agreement with previous data showing that DAB blocks learning-induced increases in extracellular lactate measured with microdialysis^7^.

Other studies suggested that lactate derived from neuronal and not astrocytic glucose, is the predominant source of activity-dependent neuronal energy substrates^26–28^. Therefore, debates have been put forward to address the disagreements^5,22–25,61,62^.

Although our data from the adult rat hippocampus engaged in long-term memory consolidation are in general agreement with the astrocyte-neuronal shuttling hypothesis proposed by Pellerin and Magistretti^63^, our conclusion does not dispute that, in other conditions and experimental models, neurons may import glucose directly via their glucose transporters and preferentially use it to support certain types of brain responses. We believe, in fact, that these debates reflect the still limited knowledge of the variety of glucose metabolism regulation engaged in different types of brain functions, stimulations, and responses, as well as age of the brain. We believe that different types of metabolic regulation mechanisms are engaged in response to different conditions and stimuli, and that conclusions drawn from certain experimental conditions and models cannot be generalized. For example, the conclusion of Patel et al.^26^, that neuronal glucose-derived pyruvate is the major oxidative fuel for activated neurons of bicuculline-treated rats, not lactate-derived from astrocytes, does not contradict that astrocyte-to-neuron lactate shuttling plays a critical role in other conditions, such as memory processes. It simply shows that epileptiform activity is coupled to neuronal glycolysis^26,64^. Obviously, learning and seizures evoke very different forms of neuronal stimulation, hence they cannot be compared.

Similarly, Diaz-Garcia et al.^27^ employed mouse hippocampal slices and in vivo whisker stimulation to measure with fluorescence biosensors the metabolic responses of individual neurons. The authors concluded that the metabolic responses they measured contradicted the astrocytic-neuronal lactate shuttling hypothesis, and instead indicate increased direct glucose consumption by neurons. However, hippocampal slices were generated from 14 to 24 postnatal mice, which very likely engaged differential regulation of glucose metabolism for at least two reasons: First, the energy metabolism regulation of the brain at this age in development is different than that of the adult^65^ (and our unpublished data). Second, acute slices as well as the whisker stimulation in vivo imaging paradigm used, are in a state of high response to injury, which is known to dramatically alter the physiological regulations of glucose metabolism^66,67^. All these deviations from learning and memory of an adult intact brain under normal physiological conditions may explain the observed shift from glycogenolysis and lactate consumption to a preferential neuronal entry and use of glucose^8,62,68^. Finally, the use of substrates to measure glucose entry should reflect the functionality of endogenous substrates^22,28^.

Hence, we suggest that, depending on the type of brain functions, age of the brain, brain region, and type of activation, distinct glucose metabolism pathways including regulation of glycogen, astrocyte-neuronal lactate coupling, pentose phosphate pathway, or glucose entering directly into neurons and undergoing neuronal oxidative glycolysis are differentially regulated and engaged. Based on our data reported here and in past studies^7,57^, we conclude that episodic memory consolidation requires rapid energy supply to neurons, but without the challenge of damaging oxidation, hence it preferentially recruits glycogenolysis and astrocytic-neuronal lactate shuttling. This lactate in neurons is, at least in part, used to generate energy for plasticity changes, including de novo mRNA translation. One other possible role of this pathway is to promote uptake and incorporation of nutrients, such as amino acids and lipids, required for synaptic morphological changes^65^.

Our data and conclusions also do not exclude the possibility that astrocytic–neuronal lactate coupling plays additional critical roles in long-term memory formation, e.g., by regulating redox or cellular signalling through direct activation of the G-protein–coupled lactate receptor GPR81/HCAR1, prostaglandin modulation, cerebral vasoconstriction and vasodilation, or other mechanisms^17,19^. Moreover, glycogen-derived glutamine, and its transport from astrocytes to neurons has been observed to be critically involved in aversive associative memory in 1-day old chicks^13^, however whether similar mechanisms are present in adult, or mammalian systems remains to be investigated.

Our results emphasize that cooperative molecular mechanisms, working across multiple cell types, i.e astrocytes and neurons, provide key biological bases for complex behavioral responses such as memory formation. Finally, our findings highlight the importance of insights into experience-induced metabolic regulation, which may yield novel hypotheses regarding the mechanisms underlying brain diseases.

## Methods

### Animals

Adult male Long–Evans rats weighing between 200 and 250 g were used. Animals were individually housed and maintained on a 12-h light/dark cycle. Experiments were performed during the light cycle. All rats were allowed ad libitum access to food and water and were handled for 3 min/d for 5 d prior any procedure. For all experiments, rats were randomly assigned to different groups. All protocols complied with the National Institutes of Health Guide for the Care and Use of Laboratory Animals and were approved by the by the Institutional Animal Care and Use Committee at New York University.

### Cannula implants and drug injection

Cannula implants were performed as described previously^7^. Briefly, rats were anesthetized with ketamine (75 mg/kg) and xylazine (10 mg/kg), and guide cannulae (C313G-SPC; 22 gauge; Plastics One) were implanted toward the dorsal hippocampus (4 mm posterior to bregma, 2.6 mm lateral to midline, 2 mm ventral to skull surface) using a stereotaxic apparatus (Kopf Instruments). Rats were administered meloxicam (3 mg/kg, sub-cutaneous, once pre-surgery), and recovered for at least 10 days before undergoing behavioral experiments. Drugs were delivered in 1.0 μl over 3 min via injection through the cannula (28 gauge, extending 1.5 mm beyond the guide) attached to polyethylene tubing (PE50) connected to a 10-μl Hamilton syringe and controlled by a microinfusion pump (Harvard Apparatus). DAB (300 pmol; Sigma), L-lactate (100 nmol; Sigma), pyruvate (100 nmol; Sigma), B3HB (72 nmol; Sigma), glucose (50 nmol; Sigma) were all dissolved in PBS (pH 7.4) and prepared fresh the day of injection. PBS injections were administered to vehicle treated and naive rats. Oligodeoxynucleotides were reverse phase cartridge-purified and purchased from Gene Link (Hawthorne, NY), were used as reported previously^7^. Cannula placement was verified at the end of the behavioral experiments, following fixation of the brains in 10% formalin. In total 40 µm coronal sections were cut through the hippocampus and examined under a light microscope. All surgeries correctly targeted the hippocampus.

### Inhibitory avoidance (IA)

IA was carried out as described previously^7,57^. The IA chamber (Med Associates) consisted of a rectangular Plexiglas box divided into a safe compartment and a shock compartment. The safe compartment was white and illuminated by a light fixture fastened to the compartment wall. The shock compartment was black and unilluminated. Foot shocks were delivered to the grid floor of this chamber via a constant current scrambler circuit. The two compartments were separated by an automatically operated sliding door. During training sessions, each rat was placed in the safe compartment with its head facing away from the door. After 10 s (s) the door automatically opened, allowing the rat access to the shock chamber. The door closed 1 s after the rat entered the shock chamber, and a brief foot shock (0.9 mA for 2 s) was administered. Latency to enter the shock compartment was taken as a measure of acquisition. The rat was then returned to its home cage. Retention tests were performed at the indicated times by placing the rat back into the safe compartment and measuring the latency to enter the shock compartment. Foot shock was not administered on the retention test, and testing was terminated at 900 s. Training and testing were performed blind to treatment conditions.

### Western blot analysis

Whole protein extracts and western blots were performed, as described previously^7^. Dorsal hippocampi were dissected rapidly in cold dissection buffer (in mM: 2.6 KCl, 1.23 sodium phosphate monobasic, 26 sodium bicarbonate, 5 kynurenic acid, 212 sucrose, 10 dextrose, 0.5 CaCl_2_, and 1 MgCl_2_), followed by homogenization in buffer containing 0.2 M NaCl, 10 mM HEPES, 2 mM EDTA, 2 mM EGTA, 0.5 mM DTT, 2 mM NaF, 1 μM microcystin, 1 mM benzamidine, and phosphatase and protease inhibitor mixtures (Sigma-Aldrich). Tissues were homogenized and centrifuged at max speed in 4°C for 30 min, after which the supernatant was removed and the remaining pellet was discarded. Protein concentrations were determined using the Bio-Rad protein assay (Bio-Rad). For Western blot analyses, equal amounts of protein (25 μg) to which 10% β-mercaptoethanol was added were resolved using 4–20% denaturing SDS–PAGE and transferred to Immunobilon-FL PVDF membranes (Millipore) by electroblotting. The membrane was dried and then blocked with 5% BSA in Tris-buffered saline + 0.1% Tween 20 (TBST, pH 7.6). Membranes were rocked gently overnight at 4 °C in primary antibody diluted in 5% BSA in TBST. Membranes were then washed and incubated with secondary antibodies for 1 h at room temperature. The following antibodies were used: anti-puromycin (1:8000, Millipore, cat #MABE343-AF647), anti-β–tubulin (1:10,000, Cel Signaling, cat#2146), anti-rabbit IRDye800CW (1:10,000, Li-Cor, cat# 926-32211) and anti-mouse IRDye680 (1:10,000, Li-Cor, cat# 926-68020). Membranes were scanned on the Li-Cor Odyssey imager under non-saturating conditions. Data were quantified using pixel intensities with the Odyssey software according to the protocols of the manufacturer (Li-Cor, Nebraska, USA). Intensities were normalized to corresponding values for β–tubulin immunoreactivity and expressed as fold changes relative to the control value.

### Immunohistochemistry

Rats were heavily anesthetized with choral hydrate (750 mg/kg), and perfused via the ascending aorta with 0.01 M phosphate-buffered saline (PBS; pH 7.4) immediately followed by ice cold 4% paraformaldehyde (PFA) in PBS. Brains were removed and post-fixed over-night in 4% PFA, and then submerged in 30% sucrose in PBS at 4 °C for at least 48 h. Thirty 30 µm thick brain sections containing the dorsal hippocampus (−2.3 mm to −4.52 from Bregma) were cut using a cryostat. Three non-serial brain slices per animal were blocked with 5% goat serum, 1% BSA, and 0.4% triton in PBS at room temperature, and then incubated in either rabbit Arc/Arg3.1 primary antibody for 24 h rocked gently at 4 ˚C (1:2000, Synaptic Systems, cat #156 003), and/or mouse anti-CaMKIIα (1:400, Millipore, cat #05-532), or mouse anti-parvalbumin (1:2500, Millipore, cat #MAB1572) for 48 h rocked gently at 4 ˚C, followed by 3 × 10 min washes in PBS at room temperature. Following the last wash, slices were incubated in secondary antibody for 2 h at room temperature, followed by 3 × 10 min washes in PBS at room temperature. Secondary antibodies used were: goat anti-rabbit IgG Alexa Fluor 568 (1:800, ThermoFisher, cat #ab175471), and goat anti-mouse IgG Alexa Fluor 488 (1:800, Invitrogen, cat #A11029). For SUnSET IHC, slices were then incubated in mouse anti-puromycin conjugated with Alexa-Fluor-647 (1:2500, Millipore, cat #MABE343 clone 12D10) for 24 h rocked gently at 4 °C followed by 3 × 10 min washes in PBS. Slices were mounted using ProLong Diamond Antifade mounting medium (ThermoFisher). Slices were imaged using a Leica SP8 confocal microscope or an Olympus VS120 fluorescent microscope. Images were analyzed and quantified with Image-J software (NIH) by an experimenter blind to treatment conditions. For SUnSET, regions of interest (ROIs) were manually drawn around CaMKIIα or PV expressing cells by an experimenter blind to treatment conditions, and ROIs were then used as masks to overlay and quantify puromycin expression in the exact same location. Arc/Arg3.1 expression analysis was performed using automated custom macro scripts. All IHC data was derived from two independent sets of experiments. Raw integrated density values were taken as measures of expression intensity.

### *In vivo* SUnSET

A protein synthesis assay was performed as previously described using the SUnSET method^40–42^. Injections of puromycin (50 μg for western blot experiments, 10 μg for IHC experiments; Sigma), puromycin and DAB (300pmol; Sigma), or puromycin and DAB and L-lactate (100 nmol; Sigma), all in 1.0 μL of PBS were administered bilaterally into the dorsal hippocampus 15 min prior to the animal being trained in IA, and rats were euthanized 2 h after training. For western blot experiments, rats were sacrificed via live decapitation, dorsal hippocampi were dissected and total extracts were prepared as described above. For SUnSET IHC experiments, rats were heavily anesthetized with choral hydrate, and IHC was performed as described above.

### Statistics and reproducibility

All experiments were repeated as indicated; *n* is the number of independent biological repeats. Numbers of independent experiments are reported in the figure legends. Data are expressed as mean ± standard error of the mean (s.e.m.) as indicated. No statistical method was used to predetermine sample size. *P* values were generated using one- or two-way analysis of variance (ANOVA), or repeated measures (RM) ANOVAs followed by Student Newman–Keuls (SNK) or Tukey post hoc tests. All tests were two-tailed and conducted using SigmaPlot (Systat software, version 11) software. Data were considered significant when ≤ 0.05. The numbers of subjects used in our experiments were the minimum to obtain statistical significance, and based on our experience with the behavioral paradigm used and in agreement with standard literature.

### Reporting summary

Further information on research design is available in the Nature Research Reporting Summary linked to this article.

## Supplementary information

Supplementary Information

Reporting Summary

## Data Availability

All data and materials produced by this study are available from the corresponding author upon request. Full Western blots and details in statistical analyses used in this study are shown in Supplementary Information.
