# Supplementary material for: Lactate from astrocytes fuels learning-induced mRNA translation in excitatory and inhibitory neurons
Source: Commun Biol. 2019 Jul 2;2:247. doi: 10.1038/s42003-019-0495-2 (PMC6606643; doi:10.1038/s42003-019-0495-2)
Supplement: Supplementary file 1 — Supplementary Information [file 42003_2019_495_MOESM1_ESM.pdf]

Supplementary Figure 1: Representative western blot membrane stained with anti-puromycin and anti-β–Tubulin pertaining to Figure 3A

Puromycin

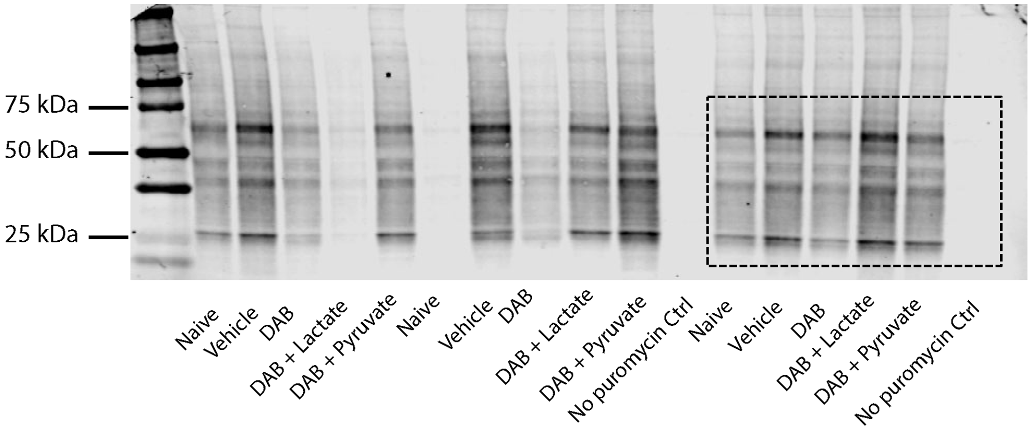

β-Tubulin

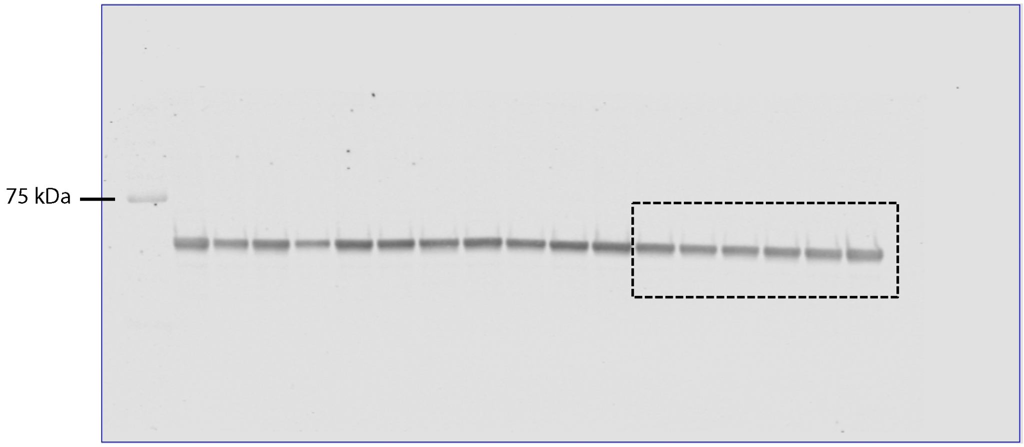

Full image of a representative blot showing data reported in Fig3A. Boxes indicate the section used in the Fig.3A representative image.

Supplementary Table 1: Statistical analyses pertaining to Figure 1

**Figure 1a**	**Mean latency (s) ± sem**		**Statistical analysis**
	Test 1	Test 2	Two-way RM ANOVA<br>Treatment: $F_{2,24}$=9.24, p=0.001<br>Time: $F_{1,24}$=6.57, p=0.017<br>Treatment X Time: $F_{2,24}$=0.78, p=0.470
Vehicle	331.11 ± 60.88	264.91 ± 71.19	
DAB	96.42 ± 26.12	46.86 ± 11.68	
DAB + Pyr	366.49 ± 63.36	219.20 ± 50.01	

**Figure 1b**	**Mean latency (s) ± sem**		**Statistical analysis**
	Test 1	Test 2	Two-way RM ANOVA<br>Treatment: $F_{1,10}$=0.32, p=0.58<br>Time: $F_{1,10}$=0.03, p=0.858<br>Treatment X Time: $F_{1,10}$=0.92, p=0.360
Vehicle	364.72 ± 56.65	412.76 ± 63.35	
Pyr	394.6 ± 70.95	323.85 ± 29.43	

**Figure 1c**	**Mean latency (s) ± sem**		**Statistical analysis**
	Test 1	Test 2	Two-way RM ANOVA<br>Treatment: $F_{3,35}$=5.52, p=0.003<br>Time: $F_{1,35}$=5.74, p=0.022<br>Treatment X Time: $F_{3,35}$=1.23, p=0.313
Vehicle	386.8 ± 54.54	291.4 ± 57.96	
DAB	100.07 ± 29.9	79.85 ± 39.41	
DAB + B3HB	380.30 ± 64.09	264.9 ± 62.97	
B3HB	289.23 ± 39.21	284.71 ± 80.34	

**Figure 1d**	**Mean latency (s) ± sem**		**Statistical analysis**
	Test 1	Test 2	Two-way RM ANOVA<br>Treatment: $F_{2,14}$=67.04, p=<0.001<br>Time: $F_{1,14}$=1.63, p=0.222<br>Treatment X Time: $F_{2,14}$=0.58, p=0.576
Veh	469.44 ± 43.76	387.31 ± 71.05	
DAB	81.81 ± 12.70	75.53 ± 21.69	
DAB + Gluc	100.48 ± 27.99	76.32 ± 16.35	

Supplementary Table 2: Statistical analyses pertaining to Figure 2

**Figure 2a**	**Mean latency (s) ± sem**		**Statistical analysis**
	Test 1	Test 2	Two-way RM ANOVA<br>Treatment: $F_{2,21}$=8.45, p=0.002<br>Time: $F_{1,21}$=1.12, p=3.01<br>Treatment X Time: $F_{2,21}$=0.21, p=0.812
SCR + Veh	335.76 ± 54.35	291.49 ± 60.16	
MCT1 AS + Veh	72.28 ± 19.63	64.07 ± 14.64	
MCT1 AS + Pyr	276.57 ± 60.87	255.79 ± 59.74	

**Figure 2b**	**Mean latency (s) ± sem**		**Statistical analysis**
	Test 1	Test 2	Two-way RM ANOVA<br>Treatment: $F_{2,32}$=10.63, p<0.001<br>Time: $F_{1,32}$=0.95, p=3.337<br>Treatment X Time: $F_{2,32}$=0.21, p=0.749
SCR + Veh	366.07 ± 53.96	119.86 ± 35.75	
MCT4 AS + Veh	119.86 ± 35.75	84.37 ± 26.13	
MCT4 AS + Pyr	327.26 ± 53.79	304.80 ± 50.27	

**Figure 2c**	**Mean latency (s) ± sem**		**Statistical analysis**
	Test 1	Test 2	Two-way RM ANOVA<br>Treatment: $F_{2,33}$=9.58, p<0.001<br>Time: $F_{1,33}$=3.01, p=0.92<br>Treatment X Time: $F_{2,32}$=0.89, p=0.422
SCR + Veh	312.45 ± 46.71	224.24 ± 46.21	
MCT1 + 4 AS + Veh	83.76 ± 21.03	70.91 ± 29.31	
MCT1 + 4 AS + Pyr	304 ± 58.80	277.14 ± 47.49	

**Figure 2d**	**Mean latency (s) ± sem**		**Statistical analysis**
	Test 1	Test 2	Two-way RM ANOVA<br>Treatment: $F_{2,15}$=0.153, p=0.860<br>Time: $F_{1,15}$=0.011, p=0.916<br>Treatment X Time: $F_{2,15}$=0.721, p=0.502
SCR + Veh	339.56 ± 34.33	373.24 ± 50.56	
SCRM + B3HB	337.13 ± 25.84	360.01 ± 41.69	
SCRM + Pyr	361.44 ± 64.14	292.51 ± 74.08	

**Figure 2e**	**Mean latency (s) ± sem**		**Statistical analysis**
	Test 1	Test 2	Two-way RM ANOVA<br>Treatment: $F_{4,51}$=15.39, p<0.001<br>Time: $F_{1,51}$=6.03, p=0.018<br>Treatment X Time: $F_{4,51}$=0.19, p=0. 944
SCR + Veh	377.27 ± 50.34	354.83 ± 57.29	
SCR + Pyr	327.02 ± 40	283.66 ± 43.64	
MCT2 AS + Veh	122.97 ± 33.15	85.44 ± 21.66	
MCT2 AS + Pyr	106.89 ± 24.63	91.98 ± 16.89	
MCT2 AS + B3HB	130.72 ± 26.47	89.88 ± 12.74	

Supplementary Table 3: Statistical analyses pertaining to Figure 3

**Figure 3a**	**Relative expression (% of naive) ± sem**	**Statistical analysis**
Untrained	100 ± 47.6	One-way ANOVA<br>Group: $F_{4,45}$=10.06, p<0.001
Veh	213.62 ± 84.20	
DAB	102.04 ± 32.64	
DAB + Lac	188.76 ± 51.30	
DAB + Pyr	183.44 ± 40.45	

**Figure 3d**	**Relative expression (% of naive) ± sem**	**Statistical analysis**
Untrained	100 ± 15.10	One-way ANOVA<br>Group: $F_{3,94}$=9.79, p<0.001
Veh	189.79 ± 17.47	
DAB	127.79 ± 8.40	
DAB + Lac	177.48 ± 10.10	

**Figure 3e**	**Relative expression (% of naive) ± sem**	**Statistical analysis**
Untrained	100 ± 13.07	One-way ANOVA<br>Group: $F_{3,102}$=23.93, p<0.001
Veh	255.79 ± 15.00	
DAB	146.76 ± 10.56	
DAB + Lac	210.83 ± 15.24	

**Figure 3f**	**Relative expression (% of naive) ± sem**	**Statistical analysis**
Untrained	100 ± 8.73	One-way ANOVA<br>Group: $F_{3,193}$=15.89, p<0.001
Veh	166.90 ± 8.97	
DAB	97.09 ± 8.72	
DAB + Lac	145.64 ± 7.93	

**Figure 3g**	**Relative expression (% of naive) ± sem**	**Statistical analysis**
Untrained	100 ± 8.32	One-way ANOVA<br>Group: $F_{3,157}$=42.57, p<0.001
Veh	216.08 ± 12.60	
DAB	64.31 ± 7.93	
DAB + Lac	211.26 ± 17.13	

Supplementary Table 4: Statistical analyses pertaining to Figure 4

**Figure 4b**	**Relative expression (% of naive) ± sem**	**Statistical analysis**
Untrained	100 ± 3.71	One-way ANOVA<br>Group: $F_{4,10}$=8.6, p=0.003
Veh	124.68 ± 1.78	
DAB	96.56 ± 5.06	
DAB + Lac	123.44 ± 6.49	
DAB + Pyr	122.93 ± 5.38	

**Figure 4c**	**Relative expression (% of naive) ± sem**	**Statistical analysis**
Untrained	100 ± 3.89	One-way ANOVA<br>Group: $F_{4,13}$=12.05, p<0.001
Veh	245.17 ± 11.21	
DAB	98.99 ± 15.97	
DAB + Lac	204.51 ± 26.12	
DAB + Pyr	212.42 ± 29.70	
