# Supplementary material for: Lactate from astrocytes fuels learning-induced mRNA translation in excitatory and inhibitory neurons
Source: Commun Biol. 2019 Jul 2;2:247. doi: 10.1038/s42003-019-0495-2 (PMC6606643; doi:10.1038/s42003-019-0495-2)
Supplement: Supplementary file 2 — Reporting Summary [file 42003_2019_495_MOESM2_ESM.pdf]

nature research

Corresponding author(s): Cristina M. Alberini, June 5, 2019

Reporting Summary

Nature Research wishes to improve the reproducibility of the work that we publish. This form provides structure for consistency and transparency in reporting. For further information on Nature Research policies, see Authors & Referees and the Editorial Policy Checklist.

Statistical parameters

When statistical analyses are reported, confirm that the following items are present in the relevant location (e.g. figure legend, table legend, main text, or Methods section).

n/a	Confirmed	
☐	☒	The exact sample size (n) for each experimental group/condition, given as a discrete number and unit of measurement
☐	☒	An indication of whether measurements were taken from distinct samples or whether the same sample was measured repeatedly
☐	☒	The statistical test(s) used AND whether they are one- or two-sided<br>*Only common tests should be described solely by name; describe more complex techniques in the Methods section.*
☐	☒	A description of all covariates tested
☐	☒	A description of any assumptions or corrections, such as tests of normality and adjustment for multiple comparisons
☐	☒	A full description of the statistics including central tendency (e.g. means) or other basic estimates (e.g. regression coefficient) AND variation (e.g. standard deviation) or associated estimates of uncertainty (e.g. confidence intervals)
☐	☒	For null hypothesis testing, the test statistic (e.g. F, t, r) with confidence intervals, effect sizes, degrees of freedom and P value noted<br>*Give P values as exact values whenever suitable.*
☒	☐	For Bayesian analysis, information on the choice of priors and Markov chain Monte Carlo settings
☒	☐	For hierarchical and complex designs, identification of the appropriate level for tests and full reporting of outcomes
☒	☐	Estimates of effect sizes (e.g. Cohen's d, Pearson's r), indicating how they were calculated
☐	☒	Clearly defined error bars<br>*State explicitly what error bars represent (e.g. SD, SE, CI)*

Our web collection on statistics for biologists may be useful.

Software and code

Policy information about availability of computer code

Data collection	Leica LAS-AF software was used to collect confocal images on a Leica SP8 confocal microscope.
Data analysis	Statistical analyses were performed using SigmaPlot (Systat software, version 11). Images were analyzed and quantified with Image-J software (National Institutes of Health, 1.52g).

For manuscripts utilizing custom algorithms or software that are central to the research but not yet described in published literature, software must be made available to editors/reviewers upon request. We strongly encourage code deposition in a community repository (e.g. GitHub). See the Nature Research guidelines for submitting code & software for further information.

Data

Policy information about availability of data

All manuscripts must include a data availability statement. This statement should provide the following information, where applicable:

- Accession codes, unique identifiers, or web links for publicly available datasets
- A list of figures that have associated raw data
- A description of any restrictions on data availability

All data and materials produced by this study are available from the corresponding author upon request.

Field-specific reporting

Please select the best fit for your research. If you are not sure, read the appropriate sections before making your selection.

☒ Life sciences ☐ Behavioural & social sciences ☐ Ecological, evolutionary & environmental sciences

For a reference copy of the document with all sections, see nature.com/authors/policies/ReportingSummary-flat.pdf

Life sciences study design

All studies must disclose on these points even when the disclosure is negative.

Sample size	We used an online statistic program (http://www.stat.uiowa.edu/~rlenth/Power/) to calculate our sample sizes. In addition, our sample sizes are similar to those generally employed in the field.
Data exclusions	No data were excluded from the study.
Replication	Behavioral experiments were replicated by multiple researchers, and multiple experiments were repeated for behavior and biochemical experiments.
Randomization	Rats were randomly assigned to treatment conditions.
Blinding	Investigators were blind to treatment conditions.

Reporting for specific materials, systems and methods

Materials & experimental systems

n/a	Involved in the study
☒	☐ Unique biological materials
☐	☒ Antibodies
☒	☐ Eukaryotic cell lines
☒	☐ Palaeontology
☐	☒ Animals and other organisms
☒	☐ Human research participants

Methods

n/a	Involved in the study
☒	☐ ChIP-seq
☒	☐ Flow cytometry
☒	☐ MRI-based neuroimaging

Antibodies

Antibodies used	Arc antibody (Synaptic Systems, cat #156 003); CaMKII-alpha antibody (Millipore, cat #05-532); Parvalbumin antibody (Millipore; cat #MAB1572); Puromycin antibody (Milliopre, cat #MABE343-AF647 and MABE343 for clone 12D10); goat anti-rabbit IgG Alexa Fluor 568 (Abcam, cat #ab175471); goat anti-mouse IgG Alexa Fluor 488 (Invitrogen, cat #A11029); anti-β–tubulin (1:10,000, Cel Signaling, cat#2146); anti-rabbit IRDye800CW (Li-Cor, cat# 926-32211) and anti-mouse IRDye680 (Li-Cor, cat# 926-68020).
Validation	Antibodies were used according to manufacturer's websites.

Animals and other organisms

Policy information about studies involving animals; ARRIVE guidelines recommended for reporting animal research

Laboratory animals	Adult (8 weeks of age), male, Long-Evans rats were used for the study.
Wild animals	The study did not involve wild animals.
Field-collected samples	The study did not involve samples collected from the field.
